# Supplementary figures and images for: Systematic proteomics analysis of lysine acetylation reveals critical features of renal proteins in kidney calculi formation
Source: PLoS One. 2026 Jan 28;21(1):e0338641. doi: 10.1371/journal.pone.0338641 (PMC12851463; doi:10.1371/journal.pone.0338641)

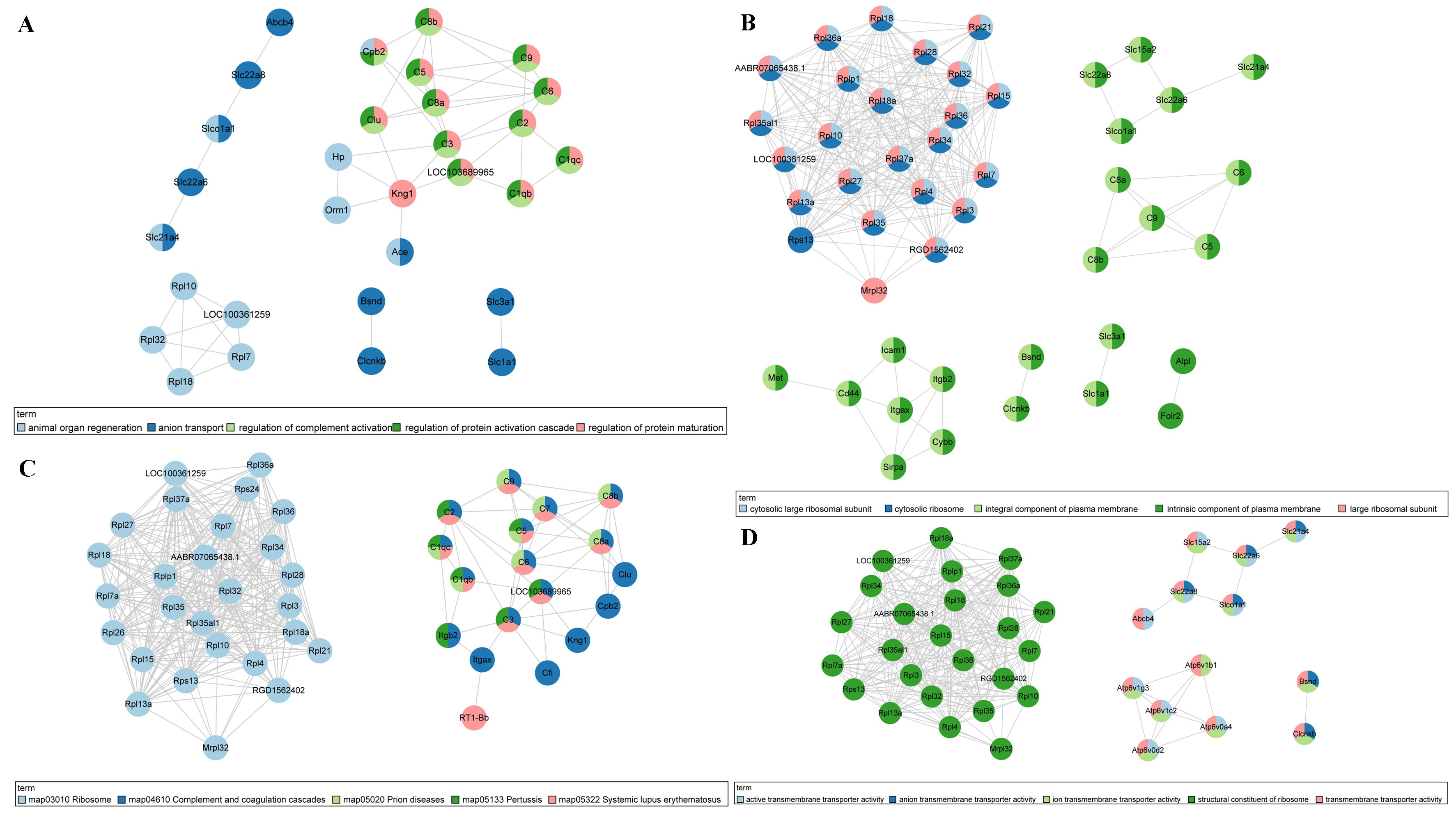

Supplement: S1 Data — S1 Fig. Interaction of differentially expressed proteins in the Top 5 pathway. A, biological process. B, cellular component. C, KEGG. D, molecular function. S2 Fig. The relationship of the differentially expressed proteins with the pathway. A, biological process. B, cellular component. C, KEGG. D, molecular function. S3 Fig. Interaction of differentially modified proteins in the Top 5 pathway. A, biological process. B, KEGG. C, cellular component. D, molecular function. S4 Fig. The relationship of the differentially modified proteins with the pathway. A, biological process. B, KEGG. C, cellular component. D, molecular function. S1 File. The detailed information of bands of interest using red boxes in Fig 1C. S2 File.The differential expressed proteins and acetylated sites. S3 File. The detailed information refers to the proteins and sites in Figs 4 and 5. (ZIP) [file pone.0338641.s001.zip › S1 Fig.tif]

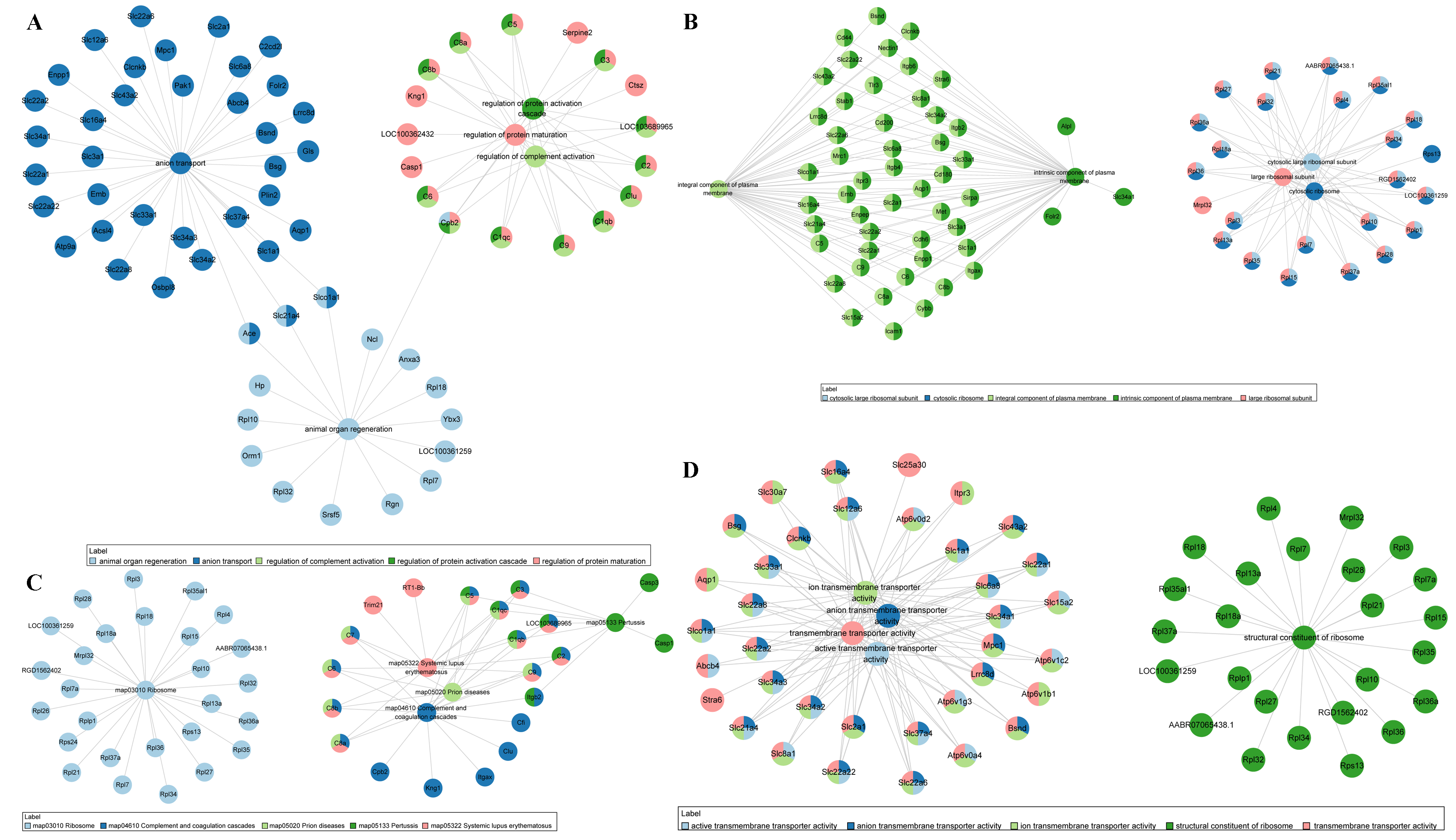

Supplement: S1 Data — S1 Fig. Interaction of differentially expressed proteins in the Top 5 pathway. A, biological process. B, cellular component. C, KEGG. D, molecular function. S2 Fig. The relationship of the differentially expressed proteins with the pathway. A, biological process. B, cellular component. C, KEGG. D, molecular function. S3 Fig. Interaction of differentially modified proteins in the Top 5 pathway. A, biological process. B, KEGG. C, cellular component. D, molecular function. S4 Fig. The relationship of the differentially modified proteins with the pathway. A, biological process. B, KEGG. C, cellular component. D, molecular function. S1 File. The detailed information of bands of interest using red boxes in Fig 1C. S2 File.The differential expressed proteins and acetylated sites. S3 File. The detailed information refers to the proteins and sites in Figs 4 and 5. (ZIP) [file pone.0338641.s001.zip › S2 Fig.tif]

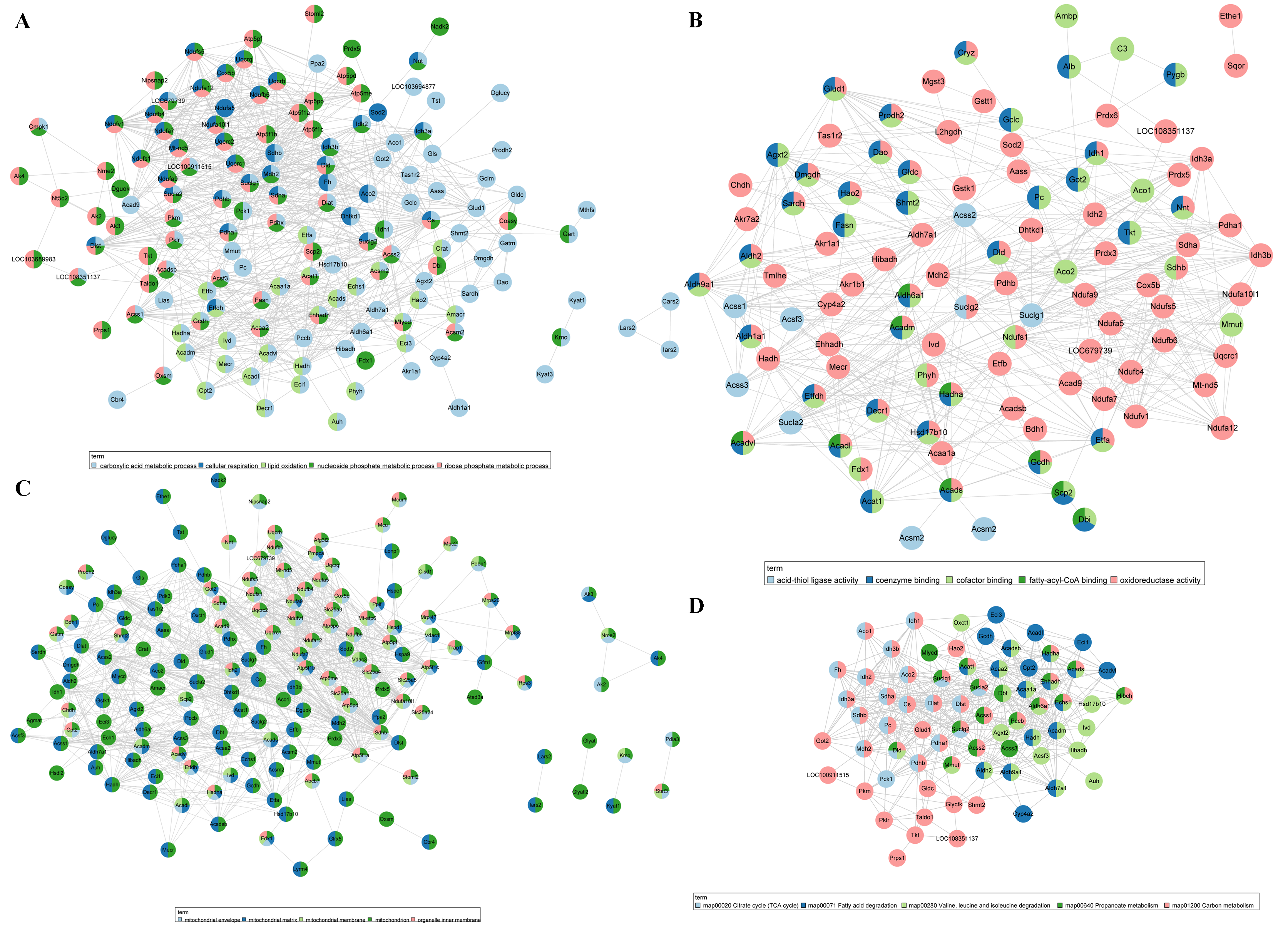

Supplement: S1 Data — S1 Fig. Interaction of differentially expressed proteins in the Top 5 pathway. A, biological process. B, cellular component. C, KEGG. D, molecular function. S2 Fig. The relationship of the differentially expressed proteins with the pathway. A, biological process. B, cellular component. C, KEGG. D, molecular function. S3 Fig. Interaction of differentially modified proteins in the Top 5 pathway. A, biological process. B, KEGG. C, cellular component. D, molecular function. S4 Fig. The relationship of the differentially modified proteins with the pathway. A, biological process. B, KEGG. C, cellular component. D, molecular function. S1 File. The detailed information of bands of interest using red boxes in Fig 1C. S2 File.The differential expressed proteins and acetylated sites. S3 File. The detailed information refers to the proteins and sites in Figs 4 and 5. (ZIP) [file pone.0338641.s001.zip › S3 Fig.tif]

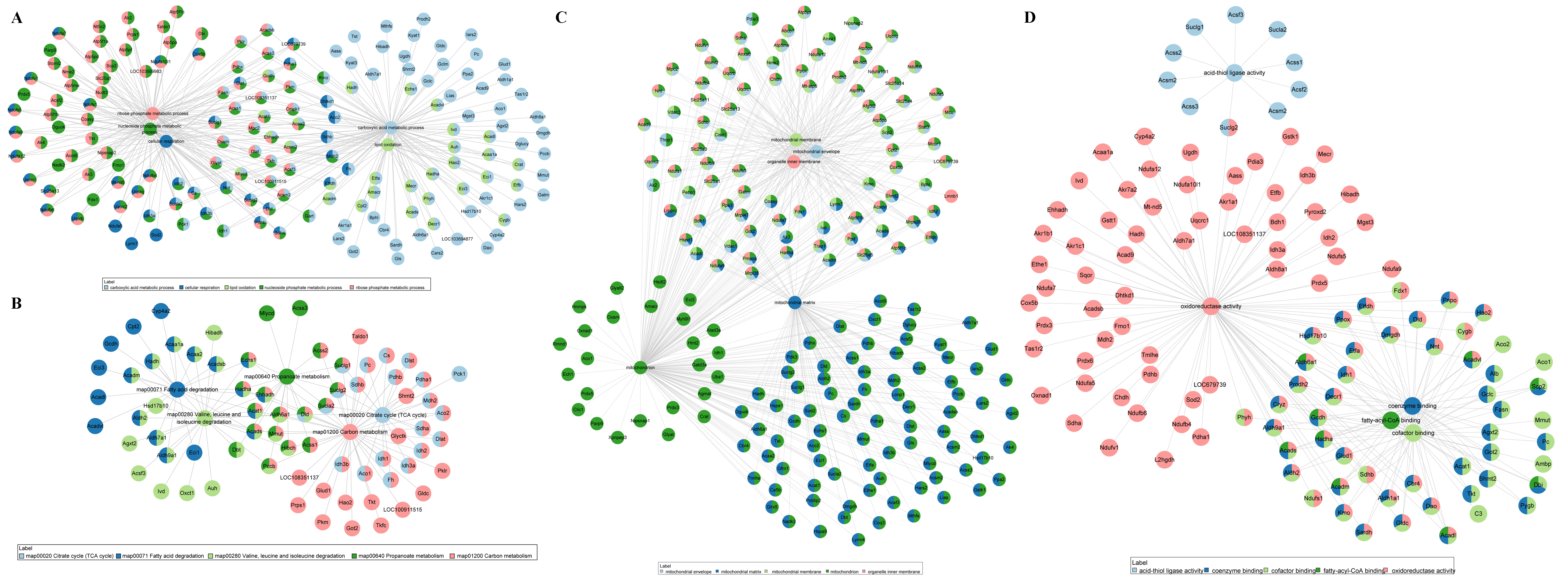

Supplement: S1 Data — S1 Fig. Interaction of differentially expressed proteins in the Top 5 pathway. A, biological process. B, cellular component. C, KEGG. D, molecular function. S2 Fig. The relationship of the differentially expressed proteins with the pathway. A, biological process. B, cellular component. C, KEGG. D, molecular function. S3 Fig. Interaction of differentially modified proteins in the Top 5 pathway. A, biological process. B, KEGG. C, cellular component. D, molecular function. S4 Fig. The relationship of the differentially modified proteins with the pathway. A, biological process. B, KEGG. C, cellular component. D, molecular function. S1 File. The detailed information of bands of interest using red boxes in Fig 1C. S2 File.The differential expressed proteins and acetylated sites. S3 File. The detailed information refers to the proteins and sites in Figs 4 and 5. (ZIP) [file pone.0338641.s001.zip › S4 Fig.tif]
