# Supplementary material for: Systematic proteomics analysis of lysine acetylation reveals critical features of renal proteins in kidney calculi formation
Source: PLoS One. 2026 Jan 28;21(1):e0338641. doi: 10.1371/journal.pone.0338641 (PMC12851463; doi:10.1371/journal.pone.0338641)
Supplement: Attachment — Submitted filename: Reponse to the Editor.docx [file pone.0338641.s003.docx]

1. PLOS ONE now requires that authors provide the original uncropped and unadjusted images underlying all blot or gel results reported in a submission’s figures or Supporting Information files. This policy and the journal’s other requirements for blot/gel reporting and figure preparation are described in detail at [https://journals.plos.org/plosone/s/figures#loc-blot-and-gel-reporting-requirements](https://journals.plos.org/plosone/s/figures#loc-blot-and-gel-reporting-requirements) and [https://journals.plos.org/plosone/s/figures#loc-preparing-figures-from-image-files.](https://journals.plos.org/plosone/s/figures#loc-preparing-figures-from-image-files.) When you submit your revised manuscript, please ensure that your figures adhere fully to these guidelines and provide the original underlying images for all blot or gel data reported in your submission. See the following link for instructions on providing the original image data: [https://journals.plos.org/plosone/s/figures#loc-original-images-for-blots-and-gels.](https://journals.plos.org/plosone/s/figures#loc-original-images-for-blots-and-gels.)

In your cover letter, please note whether your blot/gel image data are in Supporting Information or posted at a public data repository, provide the repository URL if relevant, and provide specific details as to which raw blot/gel images, if any, are not available. Email us at plosone@plos.org if you have any questions.

We've returned your manuscript to your account. Please resolve these issues and resubmit your manuscript within 21 days. If you need more time, please email the journal office at plosone@plos.org. We are happy to grant extensions of up to one month past this due date. If we do not hear from you within 21 days, we will withdraw your manuscript.

Please log on to PLOS Editorial Manager at [https://www.editorialmanager.com/pone/](https://www.editorialmanager.com/pone/) to access your manuscript. You will find your manuscript in the 'Submissions Sent Back to Author' link under the New Submissions menu. Be sure to remove your previous manuscript file if you are uploading a new file in response to these requests. After you've made the changes requested above, please be sure to view and approve the revised PDF after rebuilding the PDF to complete the resubmission process.

We are requesting these changes to comply with the PLOS ONE submission guidelines ([https://journals.plos.org/plosone/s/submission-guidelines).](https://journals.plos.org/plosone/s/submission-guidelines).) Please note that we won't send your manuscript for review until you have resolved the above requests.

Thank you for submitting your work to PLOS ONE and supporting our mission of Open Science.

R:

Dear Editor,

Thank you for your email and for providing detailed guidance on the requirements for blot and gel images. We appreciate the opportunity to revise our manuscript and are committed to adhering fully to PLOS ONE’s guidelines for reporting and submitting such data.

We have carefully reviewed the links provided regarding blot/gel reporting requirements,and the submission of original underlying images. In response to your request, we have prepared and will include the original, uncropped, and unadjusted images for all blot and gel results reported in the manuscript. These original images will be submitted in the Supplementary file 3_Original blots.

Thank you again for your assistance and for supporting open science. We look forward to your further feedback.
