# Supplementary material for: Systematic proteomics analysis of lysine acetylation reveals critical features of renal proteins in kidney calculi formation
Source: PLoS One. 2026 Jan 28;21(1):e0338641. doi: 10.1371/journal.pone.0338641 (PMC12851463; doi:10.1371/journal.pone.0338641)
Supplement: Attachment — Submitted filename: Reponse to Reviewers.docx [file pone.0338641.s004.docx]

Reviewer #1: Most of my comments have been addressed and the manuscript is clearer and more organised. However I still have a comment:

Figure 1, the authors have put together the WB panels as suggested and added boxes for the bands of interest; however the legend does not include the information that they're saying is shown in the response to reviewers: "In the figure legend, we have supplemented the detailed information about the sizes of these target bands", but the information is not in the legend. Please correct.

R: We are grateful to the reviewer for their meticulous review and for catching the inconsistency between our response and the actual figure legend. We sincerely apologize for this oversight.

We have now corrected the legend for Figure 1. The detailed information of bands of interest using red boxes was included in Supplementary file 1. Thank you again for bringing this to our attention.

Reviewer #2: Novelty: First comparative study of lysine acetylation in kidney stone pathogenesis.

Methodology: Robust proteomic and acetyl-proteomic profiling with validation.

Data: Publicly available, supporting reproducibility.

R: We are very grateful to the reviewer for their positive feedback and for highlighting the key strengths of our work. We appreciate their recognition of our study as the first comparative analysis of lysine acetylation in kidney stone pathogenesis, the robustness of our integrated proteomic and acetyl-proteomic approach with validation, and our commitment to data sharing to support reproducibility.

Reviewer #3: I appreciate the responses by the author and the updates made in the text. I still cannot read the text on figures 4,5,6 and the IF images are low resolution, making it challenging to validate the text. Once this is corrected I have no further recommendations.

R: We sincerely thank the reviewer for their time and for acknowledging our previous revisions. We are sorry to hear that the text in Figures 4, 5, 6, and the IF images remained challenging to read. Upon investigation, we believe the low resolution was an artifact introduced when the manuscript was compiled into a PDF for the review system. The original figures were created and saved at a high resolution. We apologize for this technical oversight.

We will re-export all figures (4, 5, and 6) as high-resolution PDFs or TIFFs, strictly following the journal's author guidelines. We hope that with these corrections, the figures will be perfectly clear. We appreciate your patience and look forward to your final confirmation.
